# Supplementary material for: Generation and characterisation of an Arabidopsis thaliana f3h/fls1/ans triple mutant that accumulates eriodictyol derivatives
Source: BMC Plant Biol. 2024 Feb 9;24:99. doi: 10.1186/s12870-024-04787-1 (PMC10854054; doi:10.1186/s12870-024-04787-1)
Supplement: Supplementary file 3 — Supplementary Material 3 [file 12870_2024_4787_MOESM3_ESM.pdf]

Supplementary Table S1: *A. thaliana f3h*/*fls1*/*ans* mutant *de novo* ONT genome assembly statistics.

Parameter	Value
Number of contigs	162
Maximal contig length	16,321,703 bp
Assembly size	160,571,748 bp (160.6 Mbp)
GC content	39.11 %
N50	8,170,251 bp
N90	246,021 bp
BUSCOs	95.5% complete BUSCOs<br>[Single: 88.3%. Duplicated: 7.2%]; Fragmented: 1.7%. Missing: 2.8%

Abbreviations: ONT, Oxford Nanopore Technologies; BUSCOs, Benchmarking Universal Single-Copy Orthologs

Supplementary Table S2: Oligonucleotide primers used in this work.

Gene	Name	Sequence (5'-3')	Description
F3H	MM70	GAGGTGACTAGAGACGATGAATCC	genotyping primer for the *F3H* wildtype and *tt6-2* mutant allele
	I250	AATGACGATTGGGATTTTTGTAAC	genotyping primer for the *F3H* wildtype allele
	8409	ATATTGACCATCATACTCATTGC	GABI-KAT T-DNA LB primer
FLS	UH9	CACTGAGATCTGTATGAGCCGGTACACC	genotyping primer for the *FLS* wildtype and *fls1-2* mutant allele
	RSt647	TTACACATATCAACACGTACTTTA	genotyping primer for the *FLS* wildtype allele
	RSt645	CCGGATCGTATCGGTTTTCG	RIKEN Ds-transposon primer
ANS	B078	TTCCCCTGTTTTTAAGTTTATTT	genotyping primer for the *ANS* wildtype and *tds4-4* mutant allele
	B079	AGAAAGACACAAACACATTATAAA	genotyping primer for the *ANS* wildtype allele
	RS630	GCGTGGACCGCTTGCTGCAACTCTCTCAGG	SALK T-DNA LB primer
